# Revealing the Role of CO during CO_2_ Hydrogenation on Cu Surfaces with *In Situ* Soft X-Ray Spectroscopy

**DOI:** 10.1021/jacs.2c12728

**Published:** 2023-03-14

**Authors:** Jack E. N. Swallow, Elizabeth S. Jones, Ashley R. Head, Joshua S. Gibson, Roey Ben David, Michael W. Fraser, Matthijs A. van Spronsen, Shaojun Xu, Georg Held, Baran Eren, Robert S. Weatherup

**Affiliations:** † Department of Materials, University of Oxford, Parks Road, Oxford, Oxfordshire OX1 3PH, U.K.; ‡ Center for Functional Nanomaterials, Brookhaven National Laboratory, Upton 11973, New York, United States; § Department of Chemical and Biological Physics, Weizmann Institute of Science, 234 Herzl Street, 76100 Rehovot, Israel; ∥ Diamond Light Source, Didcot, Oxfordshire OX11 0DE, U.K.; ⊥ Catalysis Hub, Research Complex at Harwell, Didcot, Oxfordshire OX11 0FA, U.K.

## Abstract

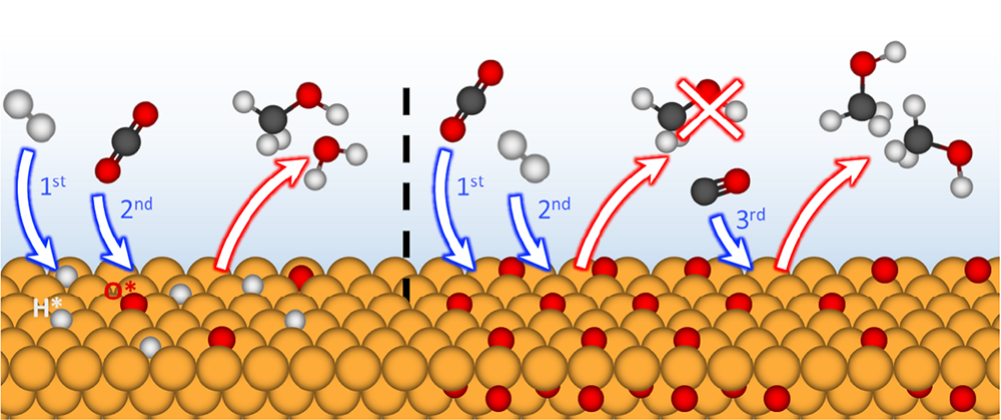

The reactions of H_2_, CO_2_, and CO gas mixtures on the surface of Cu at 200 °C, relevant for industrial methanol synthesis, are investigated using a combination of ambient pressure X-ray photoelectron spectroscopy (AP-XPS) and atmospheric-pressure near edge X-ray absorption fine structure (AtmP-NEXAFS) spectroscopy bridging pressures from 0.1 mbar to 1 bar. We find that the order of gas dosing can critically affect the catalyst chemical state, with the Cu catalyst maintained in a metallic state when H_2_ is introduced prior to the addition of CO_2_. Only on increasing the CO_2_ partial pressure is CuO formation observed that coexists with metallic Cu. When only CO_2_ is present, the surface oxidizes to Cu_2_O and CuO, and the subsequent addition of H_2_ partially reduces the surface to Cu_2_O without recovering metallic Cu, consistent with a high kinetic barrier to H_2_ dissociation on Cu_2_O. The addition of CO to the gas mixture is found to play a key role in removing adsorbed oxygen that otherwise passivates the Cu surface, making metallic Cu surface sites available for CO_2_ activation and subsequent conversion to CH_3_OH. These findings are corroborated by mass spectrometry measurements, which show increased H_2_O formation when H_2_ is dosed before rather than after CO_2_. The importance of maintaining metallic Cu sites during the methanol synthesis reaction is thereby highlighted, with the inclusion of CO in the gas feed helping to achieve this even in the absence of ZnO as the catalyst support.

## Introduction

The decarbonization of industrial processes is central to reducing global CO_2_ emissions and current societal reliance on unevenly distributed fossil fuel resources. Methanol synthesis by hydrogenation of CO_2_ offers a means of utilizing captured CO_2_ that, when combined with H_2_ produced by electrochemical water splitting, yields zero or even negative carbon emissions, as long as the required energy input comes from renewable sources. Global methanol consumption has risen dramatically in recent decades from ∼5 Mt/year in 1975 up to ∼107 Mt/year in 2021, with the majority used as feedstock for the production of important chemical derivatives.^1,2^ With an octane number of 113 and a volumetric energy density around half that of gasoline, methanol is also increasingly considered as a direct fuel for internal combustion engines and fuel cells, or indirectly as a liquid carrier for hydrogen.^3−5^

Industrial methanol synthesis is typically performed over Cu-based catalysts at 200–300 °C and 50–100 bar,^5,6^ with ∼90% of current production based on the conversion of syngas (CO/H_2_ mixture, with <20 at. % CO_2_) derived from natural gas.^5−10^ Although less common, methanol synthesis from CO_2_/H_2_ has also been industrially implemented, with further plants in development.^5^ Despite the change in reaction conditions, Cu/ZnO/Al_2_O_3_ catalysts are typically used in both cases, as well as for the low temperature water gas shift (WGS) reaction. Three key reaction pathways are thus considered in CH_3_OH synthesis from H_2_/CO_2_/CO mixtures (eqs 1, 2, and 3); CO_2_ hydrogenation, CO hydrogenation, and WGS.^11^
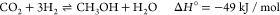
1
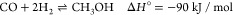
2
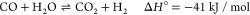
3

Isotope-labelling studies with Cu/ZnO/Al_2_O_3_ catalysts have demonstrated that CH_3_OH formation predominantly proceeds via the CO_2_ hydrogenation route (eq 1) rather than the much slower direct hydrogenation of CO (eq 2).^12,13^ This has been shown to remain the case for unsupported Cu catalysts.^12,14,15^ Even for a CO-rich feed, CO first undergoes a WGS reaction with H_2_O (eq 3) to form CO_2_ and H_2_, and then CO_2_ further reacts with H_2_ to yield CH_3_OH.^5,16^ Using a CO_2_-rich feed reduces the need for this additional WGS step; nevertheless, the presence of CO has been implicated in increasing methanol production rate in high conversion conditions.^13^ However, the beneficial role of CO remains contentious with several proposed mechanisms including the following: H_2_O removal by the WGS reaction (which otherwise kinetically inhibits methanol synthesis), regulation of adsorbed oxygen (O*) on the Cu surface, or reduction of ZnO supports to yield oxygen vacancies or Cu–Zn alloy sites.

CH_3_OH formation via the CO_2_ hydrogenation route inherently involves the liberation of O (CO_2_ + 2H_2_ ⇌ CH_3_OH + O*), which goes onto form H_2_O in the overall reaction of eq 1 (O* + H_2_ ⇌ H_2_O). Similarly, the reverse-WGS reaction (eq 3) liberates O (CO_2_ ⇌ CO + O*), with exposure of Cu surfaces to CO_2_ alone, resulting in deactivation due to O* blocking CO_2_ adsorption sites.^17^ With H_2_ present, O* can be removed by H_2_O formation, or the addition of CO to the reaction feed can shift the equilibrium, thereby regulating O* coverage. Critical to rationalizing the influence of the reaction feed on methanol productivity is a detailed account of how O* coverage and chemical state of the catalyst surface vary with the balance of reactants under realistic conditions, i.e., approaching the bar-pressure regime used industrially. However, accessing this regime with the required interface sensitivity is a significant challenge due to the dense phases on either side of the solid–gas interface,^18−20^ with the majority of reports limited either to bulk-sensitive hard X-ray absorption or pressures below a few mbar.

Herein, we investigate unsupported Cu catalysts using a combination of ambient pressure X-ray photoelectron spectroscopy (AP-XPS) and atmospheric-pressure near edge X-ray absorption fine structure (AtmP-NEXAFS) spectroscopy bridging pressures from 0.1 mbar to 1 bar. We thereby track how the O* coverage and catalyst oxidation state evolve with the composition of the reaction feed at temperatures (200 °C) and pressures (up to 1 bar) more representative of industrial methanol synthesis than prior interface-sensitive studies. For metallic Cu surfaces exposed to H_2_, the addition of a similar CO_2_ partial pressure leads to O* formation confirming CO_2_ activation, but ongoing H_2_ activation regulates the O* coverage and suppresses Cu oxidation. However, at high relative CO_2_ partial pressures, excess O* leads to Cu oxidation, poisoning the surface against CO_2_ and H_2_ activation. On removing CO_2_, a metallic Cu surface is not recovered, indicating a high kinetic barrier for H_2_ activation on Cu_2_O. Mass spectrometry (MS) measured at 1 bar corroborates Cu deactivation following CO_2_ exposure without H_2_ present: A lower H_2_O signal is observed when H_2_ is reintroduced, indicating that the CO_2_ hydrogenation and reverse-WGS reactions are suppressed. The addition of CO to the feed is able to recover metallic sites, which are most active for CO_2_ and H_2_ activation, allowing methanol generation to proceed. This highlights the important function of CO in industrial methanol synthesis from CO_2_-rich feeds, where it serves as a scavenger for adsorbed oxygen preventing saturation of the catalytically active Cu sites and making the process more robust to variations in reaction conditions.

## Experimental Methods

Three morphological variants of the Cu catalyst are used in this study: foil, thin film, and powder. Characterization of their microstructures (see Figures S1–S4) confirms that all can be considered polycrystalline, exhibiting a variety of surface orientations. The techniques used herein therefore probe an ensemble of different Cu surfaces and grain boundaries. Comparisons between the data collected with the different catalyst variants are therefore made within this context. We further note that at the pressures probed in this study, Cu can show significant restructuring.^17,21,22^

AP-XPS in the mbar pressure range was performed at the Center for Functional Nanomaterials at Brookhaven National Laboratory, USA, using a lab-based system from SPECS Surface Nano Analysis GmbH with a PHIOBOS 150 NAP hemispherical analyzer and monochromated Al Kα X-ray source (1486.7 eV).^23^ The base pressure of the chamber was ∼5 × 10^–10^ mbar. We used polycrystalline Cu foil (Alfa Aesar, 25 μm, 99.999% - metal basis) as a model catalyst, prepared by cycles of conventional *in situ* Ar^+^-ion sputtering (1 keV) and annealing (300 °C to remove implanted Ar) until the hydrocarbon signal was below the XPS detection limit. Only Cu, O, and C species were detected at the surface during the experiment. The temperature was measured using a type K thermocouple in contact with the sample surface and was stabilized at 200 °C. H_2_, CO_2_, and CO were introduced using precision leak valves, with their respective partial pressures indicated in the text below. High-resolution spectra of the Cu 2p, O 1s, and C 1s XP core-levels, Cu LMM Auger–Meitner emission, and the valence band (VB) were collected. The Cu 2p region and VB are only used for energy calibration, with the former exhibiting a negligible difference in XPS binding energy (BE) between the spectra of metallic Cu and Cu_2_O.^24^ The O 1s and C 1s core-level spectra are fitted with pseudo-Voigt functions and Shirley backgrounds within the CasaXPS software.^25^ Auger–Meitner electron spectra (AES) in the Cu LMM region are shown after subtraction of a Shirley background and the wide inelastic feature present on the low kinetic energy side. This subtraction is simply to aid in visualization of the data and has no bearing on data interpretation.

**Figure 1:**
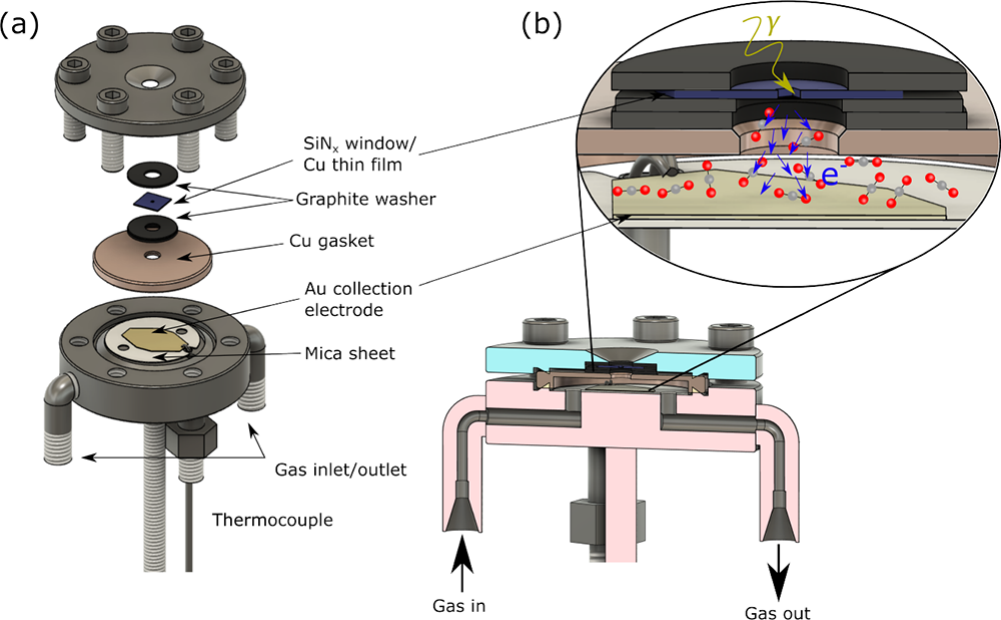
Schematic diagram of cell used for AtmP-NEXAFS spectroscopy. (a) Exploded view showing the arrangement of stacked cell components. (b) Cross section of the assembled cell. The inset shows a magnified schematic indicating the direction of incoming X-rays and generated electrons, with the cell filled with CO_2_. The electron yield signal is obtained from the current between the Cu thin film catalyst and the Au collection electrode.

AtmP-NEXAFS measurements at up to 1 bar were performed at beamline B07-C of Diamond Light Source (DLS), UK.^26,27^ A custom-designed flow cell was employed that uses an X-ray transparent silicon-nitride (SiN_*x*_) membrane (500 × 500 μm window, 100 nm thick, Silicon-rich nitride, Silson) that can withstand a >1 bar pressure difference (see Figure 1). The cell integrates a type K thermocouple to measure the temperature inside the high-pressure compartment (temperature reading thermocouple) (see Figure 1a), while an additional mineral-insulated type K thermocouple within a 310 stainless steel probe sheath is wrapped around the outside of the cell and used for resistive heating by applying a current through it (thermocouple for heating). With this design, a stable internal temperature in excess of 420 °C could be reached under vacuum (∼340 °C with 1 bar of Ar gas in the cell). The cell housing is made from 304 stainless steel vacuum flanges. An insulating mica sheet is placed on the base flange with a Au foil electrode sitting atop it. The electrode is electrically isolated from the rest of the cell and is contacted through the tip of the temperature reading thermocouple to enable the measurement of drain current. Cu (∼60 nm) atop a Cr (∼5 nm) adhesion layer was deposited onto the SiN_x_ membrane by magnetron sputtering to act as the catalyst, and faces the Au electrode within the flow cell. NEXAFS spectroscopy was performed in electron yield (EY) mode by measuring the current between the catalyst film and Au electrode when the catalyst film is under X-ray illumination.^28^ Note that AtmP-NEXAFS collected in EY mode has a probing depth of ∼10 nm for the Cu L_3_-edge collected herein, compared to ∼5 nm for the AP-XPS.^20,29^ The measurement geometry avoids simultaneous illumination of the Au electrode. The cell is sealed using a copper gasket (modified from a blank gasket by milling a cavity to accommodate the electrode without short-circuiting). A hole through the center of the gasket allows gas to reach the Cu on the SiN_x_ membrane, which is sealed against the gasket using an expanded graphite washer (Klinger).

The cell was mounted directly onto the B07-C analysis chamber, which includes a differentially-pumped beamline interface mitigating the risk of accidental gas leakage from the cell. Gases are introduced into the cell through 316 stainless steel tubing (see Figure 1b), with their flow controlled by mass flow controllers (MFCs) on the inlet and a variable pumping valve on the outlet. The pressure of the gases within the cell was monitored using a combination of capacitance (up to 100 mbar) and Bourdon-tube gauges (up to 1 bar). X-rays in the energy range of 250–2800 eV could be selected, allowing for measurements of Cu L_3_-, O K-, and C K-edges to be taken. The beamline exit slits were opened to 500 μm in the non-dispersive direction and 50 μm in the dispersive direction, which resulted in a spot size of approximately 90 μm × 100 μm.^27^ All AtmP-NEXAFS spectra are shown after subtracting a linear background fitted to the pre-edge region and post-edge normalization. Absolute energy calibration was performed using the metallic Cu catalyst obtained by H_2_ annealing (main peak set to 933.7 eV).^30^

MS measurements were performed at the Research Complex at Harwell (RCaH) using a Hiden Analytical Catlab microreactor module coupled with a quartz inert capillary continuous sampling mass spectrometer. Mass spectra of gas-phase products were recorded in a geometry consisting of a plug-flow reactor and a quadrupole MS. 150 mg of Cu powder (Alfa Aesar, −100 mesh (≤150 μm), 99%) was placed in a quartz tube (5 mm inner diameter) between two plugs of quartz wool. Cu powder was used here as a model catalyst due to the increased surface area available compared to bulk foil, thus increasing the yield of reaction products. Gas flow into the reactor was controlled using MFCs, where the total flow of gases was held at 50 standard cubic cm (sccm). Ar was used to balance the gas flow upon changing the composition of the input gas, helping to reduce fluctuations in partial pressure of each component. Atmospheric pressure was maintained within the reactor throughout the experiment. The sample was initially pretreated by introducing H_2_ (20 sccm) and annealing at 275 °C for 1 h; the Cu was determined to be fully reduced once the H_2_O signal decreased to a stable value and this was confirmed by ex situ AES following inert transfer (see Figure S5). The composition of gas flow into the microreactor was then varied sequentially using H_2_/CO_2_/CO mixtures.

## Results and Discussion

**Figure 2:**
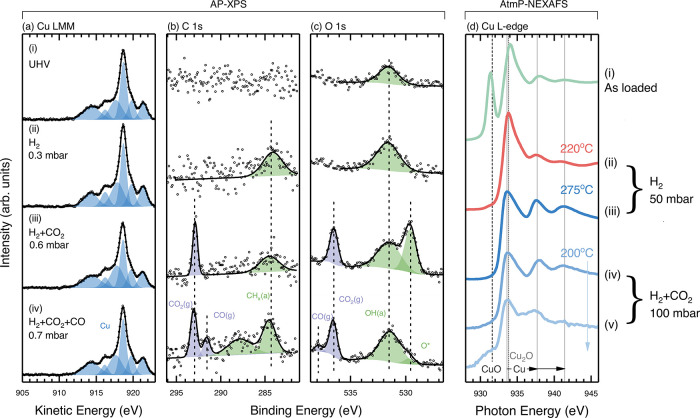
(a–c) Spectra from the Cu LMM, C 1s, and O 1s regions acquired in (i) UHV, (ii) 0.3 mbar H_2_, (iii) 0.3 mbar H_2_ and 0.3 mbar CO_2_, and (iv) 0.3 mbar H_2_, 0.3 mbar CO_2_ and 0.1 mbar CO. The temperature is fixed at 200 °C for all XP spectra. (d) AtmP-NEXAFS spectra from the Cu L_3_-edge acquired (i) for the as-entered sample, (ii) at 220 °C with 50 mbar of H_2_, (iii) at 275 °C with 50 mbar H_2_, (iv) at 200 °C with 50 mbar of H_2_ and 50 mbar of CO_2_, and (v) at 200 °C with 25 mbar of H_2_ and 75 mbar CO_2_.

**Figure 3:**
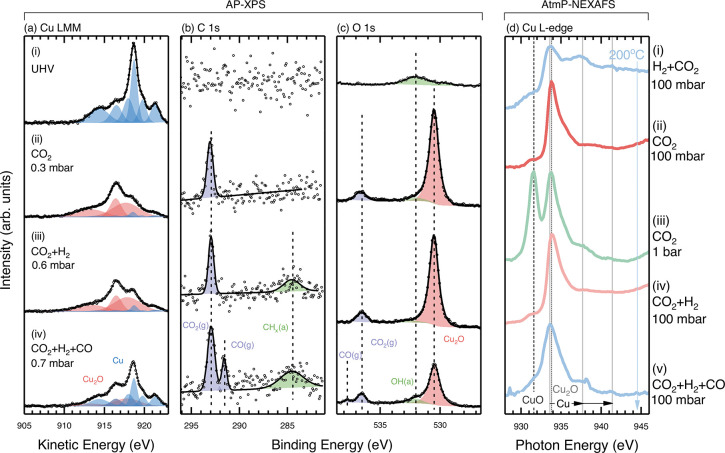
(a–c) Spectra from Cu LMM, C 1s, and O 1s regions acquired (i) in UHV, (ii) 0.3 mbar CO_2_, (iii) 0.3 mbar CO_2_ and 0.3 mbar H_2_, and (iv) 0.3 mbar CO_2_, 0.3 mbar H_2_ and 0.1 mbar CO. (d) AtmP-NEXAFS spectra from the Cu L_3_-edge. Measurements performed at (i) 25 mbar of H_2_ and 75 mbar CO_2_, (ii) 100 mbar of CO_2_, (iii) 1 bar of CO_2_, (iv) 50 mbar CO_2_ and 50 mbar H_2_, and (v) 45 mbar CO_2_, 45 mbar H_2_ and 10 mbar CO. The temperature was fixed at 200 °C for all measurements shown.

Figure 2a–c shows the collected XP spectra for the polycrystalline Cu surface during sequential exposure to different H_2_/CO_2_/CO mixtures at 200 °C. The chemical state of the Cu surface is obtained by fitting AES of the Cu LMM region shown in Figure 2a using similar line-shapes (peak position and width) as those in ref (24) to fit Cu and Cu_2_O mixtures. Peaks corresponding to metallic Cu are shown in blue, whereas those related to Cu_2_O are shown in red (only apparent in Figure 3). Gas-phase CO and CO_2_ are shown in purple and adsorbed species are shown in green.

Figure 2a(i) displays AES of the cleaned Cu surface under ultrahigh vacuum (UHV), where only peaks related to metallic Cu (blue peaks) are seen. No C species are discernable (Figure 2b(i)), indicating low levels of surface contamination, and a single broad peak is seen in the O 1s spectrum (Figure 2c(i)) at ∼531.5 eV. We tentatively assign this to hydroxylation of the surface, consistent with the binding energy position of OH commonly reported,^31,32^ which is typical for polycrystalline surfaces with high-index facets and numerous grain boundaries where trace water vapor from the measurement chamber can adsorb. However, we note that some literature studies have assigned peaks at similar positions to subsurface oxygen species, which may form during preparation of the polycrystalline foil,^33,34^ although other literature studies suggest such species to appear at lower binding energies.^35^

Upon dosing 0.3 mbar of H_2_ into the measurement environment, there are no apparent changes in the Cu LMM (Figure 2a(ii)) spectrum. A very small increase in peak intensity in the O 1s (Figure 2c(ii)) spectrum is seen. This could be from the additional H_2_O introduced upon dosing with H_2_ (contamination from gas dosing), increasing the possible OH peak; alternatively, the intensity of subsurface oxygen has previously been suggested to increase under reducing conditions.^33^ A slight increase in the intensity at ∼284.3 eV in the C 1s region (Figure 2b(ii)) is assigned to small amounts of surface hydrocarbon contamination, sometimes referred to as adventitious carbon.^31^ When only H_2_ is present in the gas phase, the Cu surface is expected to dissociate molecular H_2_ into chemisorbed hydrogen (H*),^21^ which is not readily detectable with XPS.^36^ Following the addition of 0.3 mbar of CO_2_ to the gas mixture, we still do not observe any major change in the Cu LMM spectrum (Figure 2a(iii)). However, additional peaks emerge in the C 1s (Figure 2b(iii)) and O 1s (Figure 2c(iii)) spectra. Those at ∼293.0 and ∼ 536.5 eV are attributable to CO_2_ in the gas phase.^37^ Activation of CO_2_ is confirmed by the emergence of a peak corresponding to chemisorbed oxygen (O*) at ∼529.6 eV.^17^ This indicates dissociative CO_2_ adsorption (CO_2_ ⇌ O* + CO) and/or deoxygenation of intermediate species during CO_2_ hydrogenation (e.g., H_2_COO* ⇌ O* + H_2_CO, OH* + OH* ⇌ O* + H_2_O).^38^ The lack of any discernable change in oxidation state in the AES supports the assignment of chemisorbed oxygen as opposed to lattice oxygen of oxidized Cu. A subsequent O 1s measurement 40 min later shows a similar O* intensity confirming a stable surface coverage. This suggests that O* created from ongoing CO_2_ activation reacts with H* on the Cu surface to form OH* and H_2_O rather than oxidizing the Cu surface.^39^ Note that the O 1s peak associated with H_2_O is likely to overlap with the OH* component and may mix to form hydrogen-bonded OH–H_2_O.^32,40^ Gas-phase H_2_ therefore appears to play an active role in keeping the surface metallic, with a reasonably small O* peak detected in the O 1s spectrum and no obvious Cu_2_O in the Cu LMM spectrum. At this stage, we cannot exclude the alternative explanation that competitive adsorption of hydrogen suppresses CO_2_ adsorption such that the supply of O* is insufficient for Cu oxide formation.^41^

Figure 2a(iv)–2c(iv) shows spectra after the addition of 0.1 mbar of CO into the gas mixture. The Cu LMM spectrum (Figure 2a(iv)) is once again unchanged; however, several changes are apparent in the C 1s and O 1s spectra. Alongside the peaks related to gas phase CO at ∼291.6 and ∼537.9 eV, an additional peak emerges in the C 1s region at ∼287.9 eV (Figure 2b(iv)). This could arise from oxygenated hydrocarbon contaminants,^31^ with the corresponding component in the O 1s region convoluted into the larger OH–H_2_O peak, although this could also be assigned to formate.^29^ Notably, the O* peak in the O 1s spectrum (Figure 2c(iv)) is greatly diminished in intensity relative to the other adsorbed and gas phase peaks when compared to Figure 2c(iii). This decrease is consistent with CO scavenging O* from the catalyst surface to form gas phase CO_2_, i.e., shifting the equilibrium of dissociative CO_2_ adsorption (CO_2_ ⇌ O* + CO).

To explore this behavior further, we employ AtmP-NEXAFS, where much higher gas partial pressures (>1 bar) are accessible, more closely approaching those used in industrial methanol synthesis. This technique takes advantage of the much greater attenuation lengths of photons to perform measurements through a pressure-resistant SiN_*x*_ (100 nm) membrane onto which a Cu (60 nm) catalyst film is deposited. Interface sensitivity (<10 nm) is achieved by measuring the EY signal that arises from electrons escaping the illuminated solid–gas interface.^20^

Figure 2d shows the Cu L_3_-edge NEXAFS spectra for similar gas dosing steps as used for the AP-XPS experiments but with partial pressures around two orders of magnitude higher. The as-loaded Cu film, under vacuum, exhibits two strong peaks apparent at ∼931.3 and ∼934.0 eV and two smaller features at higher photon energies (Figure 2d(i)). The first peak is attributable to CuO, which has a distinctly lower energy onset compared to Cu_2_O or metallic Cu as it involves a Cu 2p transition to empty 3d states (the Cu in CuO having a 3d^9^ configuration).^42^ Cu_2_O and metallic Cu have fully occupied 3d bands, so Cu 2p–4s transitions become dominant, giving rise to a higher absorption onset energy.^42^ Metallic Cu exhibits distinct resonances (937.6 eV and 941.3 eV) but is also expected to be more step-like in shape.^43^ Therefore, the sharp peak at ∼934.0 eV in Figure 2d(i) must originate partly from Cu_2_O, which is known to have a strong peak-like absorption onset with similar onset energy to metallic Cu,^43^ in line with previous studies.^30,44^ Therefore, the as-loaded sample shows contributions from Cu in three different oxidation states. This is attributable to the gradual oxidation of the initially metallic Cu film due to exposure to air, with Cu_2_O and CuO forming at the outermost surface whilst the bulk of the film remains metallic.

Figure 2d(ii) shows the Cu film after heating to 220 °C in 50 mbar of H_2_. All of the CuO has been reduced, as indicated by the complete absence of the peak at ∼931.3 eV. The peak at ∼934.0 eV remains, as do the resonances at higher energy. These peaks are consistent with a combination of Cu_2_O and metallic Cu, indicating that 220 °C is insufficient to fully reduce Cu_2_O to Cu in H_2_ (50 mbar). Increasing the temperature to 275 °C under the same pressure of H_2_ (Figure 2d(iii)) leads to further reduction of the surface yielding, fully metallic Cu, as seen by the step-like spectral shape with a broad absorption onset shifted to slightly lower energy (∼933.7 eV), and the relatively stronger fine structure peaks (∼937.6 and ∼941.3 eV).

After fully reducing the surface to a metallic state, the temperature was lowered to 200 °C, and CO_2_ was introduced into the chamber matching the partial pressure of H_2_ (50 mbar of CO_2_ + 50 mbar H_2_). Figure 2d(iv) shows negligible changes to the spectrum acquired, which retains its metallic line shape, confirming that the findings of Figure 2a–c hold at higher pressures. On increasing the partial pressure ratio to 75 mbar CO_2_ + 25 mbar H_2_ (Figure 2d(v)), the Cu remains predominantly metallic. However, there is a slight weakening of the fine structure peaks and the emergence of a small peak at ∼931.5 eV, indicating that a small amount of Cu_2_O and CuO is formed. Therefore, the ratio of H_2_ and CO_2_ gas is important, with further increases in CO_2_ partial pressure expected to yield more oxidation of the Cu surface. Note that in addition to the Cu L_3_-edge, C K-edge and O K-edge were also acquired during the AtmP-NEXAFS experiment; however, gas phase peaks from CO_2_ and CO (after inclusion in the gas mixture) dominate these spectra.

Figure 3 shows the results of similar AP-XPS and AtmP-NEXAFS experiments but where the order of CO_2_ and H_2_ introduction is reversed. Prior to the AP-XPS measurements in Figure 3a–c, the Cu surface was cleaned by sputtering and annealing; on the other hand, for AtmP-NEXAFS, the Cu surface was untreated, with the measurements continuing directly from Figure 2. Figure 3a(i) shows the Cu LMM spectrum immediately following the surface preparation. This matches the spectrum seen in Figure 2a, displaying only the features associated with metallic Cu. Similarly, no hydrocarbon contamination is seen in the C 1s spectrum (Figure 3b(i)), and the hydroxyl peak is still visible in the O 1s region (Figure 3c(i)) due to small and unavoidable water vapor contamination in the AP measurement chamber. The slightly higher binding energy of this O 1s peak (∼532.0 eV) compared to Figure 2c(i) (∼531.5 eV) corresponds more closely to surface rather than subsurface oxygen species.^32,34^ A small shoulder peak is also seen here at ∼529.5 eV. This may be attributable to a small amount of Cu_2_O (not resolvable in the AES), although its lower binding energy and larger FWHM (∼0.5 eV wider) compared to the other Cu_2_O peaks indicates that it is more likely related to O* at the surface.^45,46^

Once 0.3 mbar CO_2_ is dosed into the chamber (Figure 3a(ii)), the Cu LMM spectrum changes significantly, accompanied by the emergence of an intense peak at ∼530.5 eV in the O 1s region (Figure 3c(ii)). This indicates Cu_2_O formation arising from CO_2_ dissociation on the surface. Lattice oxygen in Cu_2_O was previously reported to produce a peak at around 530.1–530.4 eV;^24,31,45,47^ the small shift in position seen here is within experimental error and could be due to variations in binding strength across different crystal orientations on the polycrystalline sample.^48^ From the AES, the percentage of Cu_2_O can be estimated as ∼85% of the detected signal. Considering the roughly 1 nm inelastic mean free path (IMFP) of the Auger–Meitner electrons, we can estimate the nominal thickness of the oxide layer on the surface to be at least ∼1 nm. Such prominent oxidation of the surface and a few subsurface layers is again consistent with dissociative adsorption of CO_2_,^17,39,49^ which supplies O* that oxidizes the initially metallic Cu. CO, which also forms due to CO_2_ dissociation, is expected to desorb immediately to the gas phase at 200 °C.^50^ Thus, no features attributable to adsorbed CO or CO_2_ are expected in the C 1s and O 1s spectra of Figure 3b,c(ii), although gas phase CO_2_ is still apparent. Similar to the O 1s spectra obtained in vacuum, the surface appears to be partly hydroxylated.

The addition of 0.3 mbar H_2_ to the 0.3 mbar CO_2_ does not result in any considerable change in the oxidation state of Cu, with an ∼85% oxide ratio still obtained from the Cu LMM spectrum (Figure 3a(iii)) and the relatively intense peak at around ∼530.5 eV persisting (Figure 3c(iii)). Moreover, the addition of H_2_ does not change the intensity ratio between the O 1s peaks arising from gaseous CO_2_ and lattice Cu_2_O. A hydrocarbon peak does, however, emerge in the C 1s region (Figure 3b(iii)), as also seen when H_2_ is present in Figure 2b.

Only after the introduction of 0.1 mbar CO into the gas mixture (Figure 3a(iv)) does the oxide-to-metallic ratio obtained from the Cu LMM spectrum reduce to ∼35%, accompanied by a reduction in the intensity of the Cu_2_O peak at ∼530.5 eV (Figure 3c(iv)). The O 1s XPS intensity ratio between lattice oxygen and oxygen in gas-phase CO_2_ drops to ∼60% of that without CO present. We thus suggests that CO plays an active role in reducing Cu_2_O, making more metallic adsorption sites available by scavenging oxygen on the surface. The XP spectra presented here were acquired within 1 h of the introduction of CO, with longer exposures of >2 h at the same temperature eventually leading to the recovery of metallic Cu to a large extent (data not shown). We note that the peaks attributed to hydrocarbons and hydroxyl groups persist when CO is dosed; however, oxygenated hydrocarbon contaminants are not discernable. Gas phase CO is clearly seen in both the C 1s and O 1s spectra in addition to gas phase CO_2_ (Figure 3b(iv),c(iv)).

Figure 3d shows the Cu L_3_-edge NEXAFS following on from the experiment displayed in Figure 2d(v), i.e., Figure 2d(v) and Figure 3d(i) are the same spectrum. In order to simulate the gas dosing protocol used for AP-XPS in Figure 3(ii), the flow of H_2_ into the cell was stopped, leaving only CO_2_ at a pressure of 100 mbar (Figure 3d(ii)). A fairly rapid change from metallic Cu to Cu_2_O is observed (measurements performed ∼30 mins apart), with a small contribution from CuO also apparent.

Following this initial change, the pressure of CO_2_ was increased to 1 bar (Figure 3d(iii)), where we see significant CuO formation not seen at lower CO_2_ pressures. This can be understood as the increase in partial pressure of CO_2_ shifting the chemical equilibrium in favor of increased CO_2_ dissociation, thereby increasing the supply of O*, which feeds CuO formation. This result highlights the importance of approaching realistic reaction conditions (in this case, high pressures) when studying catalytic reactions, as even under AP conditions, the catalyst state can differ from that seen at higher pressures.

After adding H_2_ (50 mbar) back into the gas mixture and returning to a total pressure of 100 mbar (Figure 3d(iv)), we see the removal of most CuO with the Cu_2_O peak again dominating; however, no features of metallic Cu are apparent. Only after adding CO into the gas mixture (45 mbar CO_2_, 45 mbar H_2_, and 10 mbar CO) does the line shape become less asymmetric, and the fine structure peaks of metallic Cu emerge (Figure 3d(v)), indicating partial reduction of the surface. As observed in Figure 3a(iv), at this relatively low ratio of CO, the surface does not become fully metallic; however, some reduction of the surface by CO is seen. CO is thus confirmed to increase the number of metallic sites, which are most catalytically active for H_2_ and CO_2_ dissociation.

By comparing Figure 2(iv) and Figure 3(iv), our AP-XPS and AtmP-NEXAFS results reveal a significant difference in catalyst oxidation state depending on the order of gas dosing for otherwise identical reaction conditions (1:1 mixture of CO_2_:H_2_ at either a total pressure of 0.6 mbar (AP-XPS) or 100 mbar (AtmP-NEXAFS)). If thermodynamic equilibrium is reached, then the final state should be independent of the direction of the approach. Here, we find that the Cu surface remains predominantly metallic when H_2_ is dosed prior to CO_2_ (Figure 2(iv)), even when relatively CO_2_-rich conditions are reached (3:1, Figure 2(v)). However, when CO_2_ is introduced first, the Cu surface oxidizes toward CuO, with the subsequent addition of H_2_ only leading to partial reduction to predominantly Cu_2_O (Figure 3(iv)). Even pure H_2_ (50 mbar) at 220 °C does not yield a fully metallic Cu surface, with higher temperatures (275 °C) needed to achieve this (see Figure 2d(iii)), indicating a large kinetic barrier for H_2_ dissociation on Cu.^51,52^ Loosely packed metallic Cu surfaces, on the other hand, dissociate H_2_ at a reasonable rate even at room temperature.^21^ In this context, when H_2_ is dosed first, ongoing H_2_ dissociation on the metallic Cu presumably scavenges O* from CO_2_ activation to form OH* and H_2_O, thereby preventing Cu oxidation.^39,53−55^ Given that Cu is found to remain reduced across a wide pressure range (up to 100 mbar) and the presence of O* in AP-XPS measurements confirms ongoing CO_2_ activation, the suppression of Cu oxidation by competitive hydrogen adsorption can be largely excluded.

Once the Cu surface is oxidized, the rate of H_2_ dissociation becomes very low and insufficient to recover a metallic surface. However, our results confirm that the metallic character is at least partially recovered by the addition of CO, which is more effective at reducing Cu_2_O compared to H_2_. Dissociation of H_2_ is critical to methanol synthesis, and our results here point toward an important role for CO in the gas mixture: as an oxygen scavenger to maintain metallic Cu sites. Maintaining these sites allows both H_2_ and CO_2_ dissociation to proceed, especially as CO_2_ activation tends to rapidly oxidize and deactivate the surface unless O* is continuously removed.^17^

We have so far considered soft X-ray spectroscopy measurements to follow changes in the chemical state of the catalyst surface with gas dosing. To complement these studies, MS was used to observe how the reaction products vary with the order of H_2_ and CO_2_ dosing. We focus here on the H_2_O signal (a product of eqs 1 and 3), noting that the yield of CH_3_OH is small for unsupported Cu, and the setup used is not well-optimized for CH_3_OH detection (see Figure S1).

**Figure 4:**
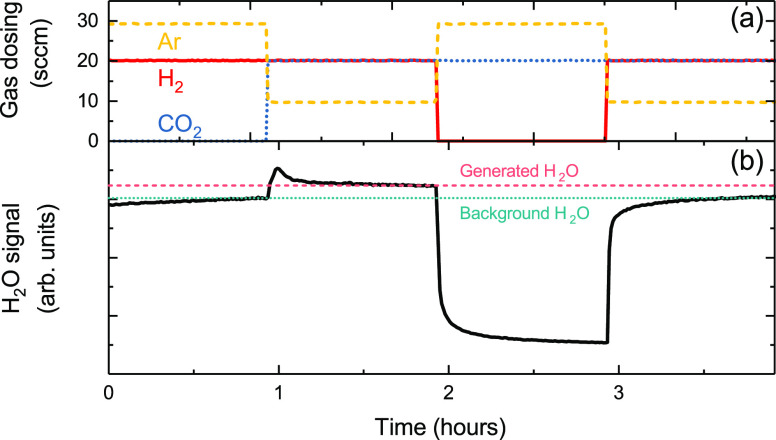
Time evolution of H_2_O mass signal for different reaction steps. (a) Flow rates of gases dosed during the reaction. The gas dosing steps proceeded as follows: H_2_ (20 sccm), H_2_ (20 sccm) and CO_2_ (20 sccm), CO_2_ (20 sccm), and finally H_2_ (20 sccm) and CO_2_ (20 sccm). Ar gas was used to balance the chamber pressure and maintain a constant total flow of 50 sccm. (b) H_2_O signal during the reaction. The zero of the time scale is set to when the temperature reached 200 °C and the pressure is maintained at 1 bar throughout. At t = 0 h, the Cu is expected to be metallic after having been reduced in H_2_ at 275 °C.

Figure 4 shows the H_2_O signal generated from the Cu powder over time. Prior to this, the Cu was reduced in H_2_ at 275 °C to yield metallic Cu, as confirmed by the AtmP-NEXAFS and further ex situ characterization (see Figures S4 and S5). During the first hour, a stable H_2_O signal is observed while dosing H_2_, corresponding to a background level of H_2_O arising from residual species from the gas lines/reactor walls. For the second hour, CO_2_ is introduced alongside H_2_. There is a clear initial increase (peak) in the H_2_O signal, which decays and stabilizes at a higher level than the background signal. The initial increase is characteristic of a transient state brought about by the surface reacting to the change in conditions. The higher level at which the H_2_O signal equilibrates is attributable to H* (from H_2_ dissociation) on the Cu surface reacting with O* and other reaction intermediates (from CO_2_ activation) to form H_2_O as part of the CO_2_ hydrogenation (eq 1) and/or reverse-WGS reactions (eq 3).^38,56^ This can also explain the transient behavior, with the steady-state H* coverage stabilized during H_2_ exposure serving as a reservoir for reaction with O* when CO_2_ is introduced, resulting in an elevated H_2_O signal until a new steady-state H* coverage is reached. This further supports the arguments presented above that the reaction of H* with the O* generated by CO_2_ activation is primarily responsible for maintaining the Cu surface in a metallic state rather than the suppression of CO_2_ adsorption by competitive H* adsorption.

In the third hour of the experiment, H_2_ is removed from the gas mixture, and as expected, the H_2_O signal drops to a much lower level. During this exposure to CO_2_ alone, the surface of the Cu will be oxidized, as confirmed by the AtmP-NEXAFS measurements of Figure 3d(ii, iii). When H_2_ is reintroduced into the gas mixture for the fourth hour, the H_2_O signal returns to the background level seen when only H_2_ is present, and no initial peak associated with a transient state is observed. The lack of additional H_2_O formation is consistent with observations from the earlier spectroscopy measurements: a large kinetic barrier to H_2_ dissociation exists on Cu_2_O, meaning that it is not reduced to metallic Cu by H_2_ addition at 200 °C. It also indicates that CO_2_ hydrogenation and reverse-WGS reactions are heavily suppressed compared to when H_2_ is dosed onto metallic Cu prior to the CO_2_. Although the addition of CO is expected to recover the catalytically active metallic sites, this will also cause a shift in the equilibrium of eq 3 suppressing H_2_O formation by WGS. Indeed, a slight drop in the H_2_O mass signal is observed on CO addition (see Figure S6), consistent with CO providing an additional pathway for O* removal.

**Figure 5:**
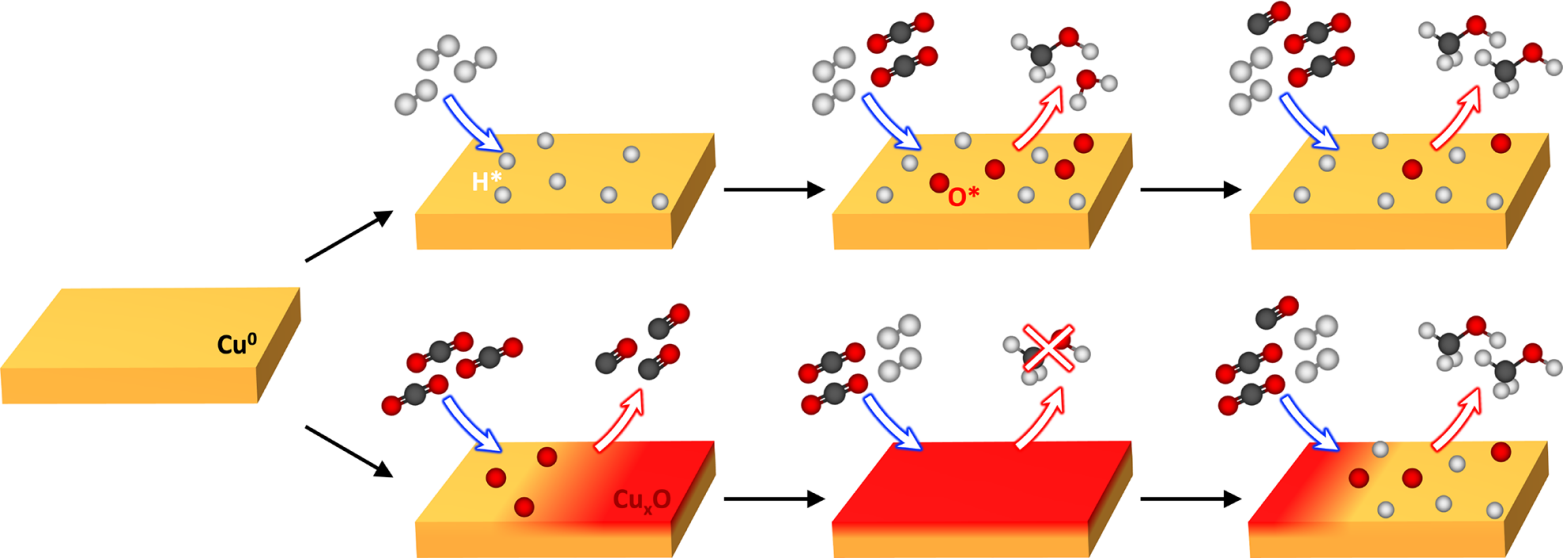
Illustrative diagram of the proposed reaction pathways during exposure of Cu to H_2_, CO_2_ and CO. The top row corresponds to the gas dosing order in Figure 2, i.e., H_2_ first, while the bottom row corresponds to the dosing order in Figure 3, i.e., CO_2_ first.

Figure 5 summarizes the main reaction pathways revealed by this study when H_2_/CO_2_/CO gas mixtures react on Cu surfaces. The ratio of H_2_ to CO_2_ and the order in which they are introduced can significantly alter the catalyst state and its activity toward methanol synthesis reactions.

Starting from an initially metallic catalyst, we find that when H_2_ is used as the initial reactant gas, the Cu surface is maintained in a metallic state, with hydrogen acting to remove any residual atomic oxygen from the Cu surface. When CO_2_ is introduced into the gas mixture, its activation on the Cu surface is observed through the emergence of chemisorbed atomic oxygen, but without formation of a distinct Cu oxide phase (apart from at high CO_2_:H_2_ ratios when Cu, Cu_2_O, and CuO are found to coexist). This behavior can be accounted for by the ongoing supply of H* through H_2_ dissociation, which removes O* produced from CO_2_ activation in the form of H_2_O vapor, thereby preventing it from oxidizing the surface. This is supported by MS (Figure 4) where the H_2_O signal increases when CO_2_ is added to the gas mixture. This confirms CO_2_ and H_2_ activation on the metallic Cu surface, which are both critical to the CO_2_ hydrogenation and reverse-WGS reactions that occur under methanol synthesis conditions. Previous literature studies indicate a clear correlation between CO_2_ activation and Cu coordination, following the order Cu(110) > Cu(100) > Cu(111) for the low-index surfaces, with reported activation energies for CO_2_ dissociation of 0.64–0.67 eV on Cu(110),^49,57^ 0.83–0.96 eV on Cu(100),^58,59^ and 0.93–1.33 eV on Cu(111).^49,60^ Steps are found to be preferential sites for CO_2_ dissociation, and their presence may account for some of the lower barriers obtained from experimental studies.^58,60^ Activation energies for H_2_ dissociation are generally much lower and follow the same order for the low-index surfaces: 0.28 eV on Cu(110),^48^ 0.51 eV on Cu(100),^48^ 0.54 eV on Cu(111).^61^ Therefore, although our samples herein are polycrystalline, these trends are fully consistent with the behavior we observe with the more rapid dissociation of H_2_ on metallic Cu able to remove excess O* produced from CO_2_ activation.

When the ratio of CO_2_:H_2_ is increased, a small amount of oxide formation is observed, as in Figure 2d(v). This oxidation can be rationalized by the amount of O* increasing relative to H* on the surface, such that some excess O* does not react with H* to form H_2_O and is thus available to oxidize Cu. The addition of CO has little effect on the chemical state of the already metallic catalyst; however, the concentration of atomic oxygen is significantly reduced on the Cu surface, highlighting the role of CO as an oxygen scavenger.^62^ Thus, while CO acts as an oxygen scavenger to maintain a catalytically active surface, this is less crucial when H_2_ is dosed prior to CO_2_.

On the other hand, if CO_2_ is used as the initial reactant gas, then significant oxidation of the Cu surface is observed (2Cu + O* ⇌ Cu_2_O), attributable to O* provided by CO_2_ dissociation. CO_2_ is found to adsorb as CO_2_^δ−^ on Cu(111) at low pressures (0.01–1 mbar) and room temperature,^17,49^ but on more active Cu (100), Cu(110), and stepped surfaces, CO_2_ dissociation is observed through the emergence of chemisorbed oxygen^63,64^ and is even seen on Cu(111) as pressure and temperature are increased.^49^ However, O* coverage blocks further CO_2_ adsorption on the catalyst surface leading to “self-poisoning”.^17,39,49^ Although O* coverages of >0.5 monolayers have been observed on Cu(100) during CO_2_ exposure through the breakup of the surface into nanoclusters, the Cu subsurface remains predominantly metallic at room temperature.^17^ The greater extent of catalyst oxidation observed on polycrystalline Cu herein is attributable to the higher temperature during CO_2_ exposure, with the higher oxygen diffusivity presumably facilitating Cu_2_O formation.

At higher CO_2_ pressures of 100 mbar and above, we observe the emergence of CuO at the surface (Cu_2_O + O* ⇌ 2CuO), which is an appreciable component at 1 bar. On lowering the CO_2_ pressure and introducing H_2_, the catalyst reduces again to predominantly Cu_2_O, but further reduction to metallic Cu is not observed. We suggest that this is due to a large kinetic barrier for H_2_ dissociation on Cu_2_O (as previously discussed).^51^ Indeed, calculations of H_2_ dissociation on oxygen covered Cu surfaces obtained activation energies of 1.06 eV on O(2 × 2)/Cu(100).^65^ This large barrier is also apparent from our AtmP-NEXAFS measurements in Figure 2d(ii, iii), where heating to 220 °C in H_2_ (50 mbar) is not sufficient to fully reduce the Cu_2_O surface, which only becomes metallic after heating to 275 °C. Our MS results (Figure 4) are also consistent with this, with a similar H_2_O signal observed when H_2_ is added after CO_2_ dosing to when only H_2_ is dosed. We note that these findings are broadly consistent with prior DFT calculations suggesting that CuO is easier to reduce than Cu_2_O in the presence of H_2_.^66^

Although the addition of H_2_ after CO_2_ does not reduce the surface beyond Cu_2_O, we find that further reduction can be achieved by the addition of CO (Cu_2_O + CO ⇌ 2Cu + CO_2_). CO is thus conclusively shown to behave as an oxygen scavenger, yielding metallic sites that are more catalytically active for CO_2_ activation and H_2_ dissociation. An overall activation energy of 0.26 eV for the reduction of Cu_2_O with CO is reported based on thermogravimetric analysis,^67^ while single-crystal AP-XPS studies have determined activation energies for the removal of preadsorbed O on Cu(111) as 0.24 eV, Cu(100) as 0.29 eV, and Cu(110) as 0.51 eV.^46^ These are generally well below the corresponding activation barriers for CO_2_ dissociation, and while we consider polycrystalline catalyst surfaces herein, this is nevertheless consistent with our observation that a small addition of CO to the reactant feed is sufficient to maintain metallic Cu by the rapid removal of excess O*. Maintaining metallic Cu sites is critical to achieving methanol generation at a significant rate, with an increase in the number of these sites shown to increase activity toward methanol formation.^68^ From eq 3, it is also clear that introducing CO shifts the equilibrium making reverse-WGS less favorable such that more CO_2_ is directly converted to CH_3_OH rather than being converted into CO. Thus, our results demonstrate the importance of using CO in the gas feed for methanol synthesis from CO_2_ and H_2_ to prevent Cu catalyst deactivation by maintaining metallic sites across a wider range of conditions, including at high CO_2_:H_2_ ratios.^17^

While our focus herein has been the surface of unsupported Cu catalysts, industrially, ZnO is typically used to support Cu for methanol synthesis. There remains significant debate over the exact nature of the interaction between Cu and ZnO that leads to improved methanol yield.^69−72^ However, it is well accepted that in the presence of ZnO, the Cu remains metallic during methanol synthesis.^70,72^ Our results highlight the importance of Cu remaining metallic in order to activate H_2_ as well as CO_2_, and spillover of H* to ZnO has been implicated in the formation of reactive intermediates.^70^ There has been much recent discussion on the nature of reduced Zn species and the intermediates responsible for the promotional effect of ZnO supports; for example, the formation of a CuZn alloy is often considered, but this requires highly reducing conditions.^69,71,72^ The inclusion of CO has been shown to help maintain such a CuZn alloy,^72^ playing a similar role of oxygen scavenger as observed herein, albeit promoting the reduction of different species (since the presence of Zn already maintains metallic Cu). Hence, the precise role of CO may depend critically on the catalyst composition used.

## Conclusions

In summary, we have shown how the surface chemical state and absorbates present on Cu vary with the order of gas dosing during CO_2_ and H_2_ exposure at temperatures typically used for methanol synthesis. We note that through our complementary AP-XPS and AtmP-NEXAFS study, the understanding developed is extended to the atmospheric pressure regime while still providing surface-sensitive information through soft X-ray spectroscopic techniques. This combined approach proves a practical avenue for studying catalytic reactions at industrially relevant gas pressures. We find that the Cu surface remains metallic in the presence of CO_2_ and H_2_, when H_2_ is dosed first. CuO is observed at high CO_2_ ratios; however, Cu retains the significant metallic character needed for ongoing CO_2_ and H_2_ activation. When CO_2_ is dosed prior to H_2_, the Cu surface readily oxidizes to Cu_2_O, with significant CuO formation at high CO_2_ pressures. The introduction of H_2_ only returns the surface to Cu_2_O with further reduction suppressed due to a high kinetic barrier for H_2_ dissociation. The addition of CO to the gas feed is found to scavenge oxygen from the Cu surface, thus making metallic Cu sites available for CO_2_ and H_2_ activation. Hence, even though CO_2_ has been established as the carbon source for methanol generation,^12^ this study demonstrates the importance of including CO in the reactant mixture. This contributes to a process that is more robust to variations in reaction conditions, particularly given that large kinetic barriers for certain processes (e.g., H_2_ activation) can lead to effective catalyst deactivation if the desired oxidation state is lost. Our results highlight the importance of studying these phenomena at pressures close to realistic industrial conditions, as this can alter both the equilibrium and reaction kinetics.

## Supporting Information Available

Microstructural and chemical characterization of the Cu catalyst variants; time evolution of H_2_O and CH_3_OH MS signals for different reaction steps (PDF)

## Notes

The authors confirm that the data supporting the findings of this study are available within the article and its supplementary materials.

## Abbreviations

AES: Auger–Meitner electron spectroscopy
AP: ambient pressure
EBSD: electron backscatter diffraction
MS: mass spectrometry
NEXAFS: near edge X-ray absorption fine structure
SEM: scanning electron microscopy
WGS: water gas shift
XAS: X-ray absorption spectroscopy
XPS: X-ray photoelectron spectroscopy
