# Supplementary material for: Revealing the Role of CO during CO2 Hydrogenation on Cu Surfaces with In Situ Soft X-Ray Spectroscopy
Source: J Am Chem Soc. 2023 Mar 14;145(12):6730–40. doi: 10.1021/jacs.2c12728 (PMC10064333; doi:10.1021/jacs.2c12728)
Supplement: Supplementary file 1 — ja2c12728_si_001.pdf [file ja2c12728_si_001.pdf]

Supporting Information: Revealing the Role of CO during CO_2 Hydrogenation on Cu Surfaces with *In Situ* Soft X-ray Spectroscopies

Jack E. N. Swallow[a], Elizabeth S. Jones[a], Ashley R. Head[b], Joshua S. Gibson[a], Roey Ben David[c], Michael W. Fraser[a], Matthijs A. van Spronsen[d], Shaojun Xu[e], Georg Held[d], Baran Eren*[c], Robert S. Weatherup*[a,d]

[a] Department of Materials,
University of Oxford
Parks Road, Oxford, Oxfordshire, OX1 3PH, United Kingdom

[b] Center for Functional Nanomaterials
Brookhaven National Laboratory
Upton 11973, New York, United States

[c] Department of Chemical and Biological Physics
Weizmann Institute of Science
234 Herzl Street, 76100 Rehovot, Israel

[d] Diamond Light Source,
Didcot, Oxfordshire OX11 0DE, United Kingdom

[e] Catalysis Hub,
Research Complex at Harwell,
Didcot, Oxfordshire, OX11 0FA

E-mail: baran.eren@weizmann.ac.il, robert.weatherup@materials.ox.ac.uk

KEYWORDS: Copper • CO2 hydrogenation • ambient pressure • atmospheric pressure • x-ray spectroscopy • methanol • CO

Microstructural Characterization of Cu catalysts

Three morphological variants of Cu catalyst are used herein: Cu foil (used for AP-XPS), Cu thin film (used for AtmP-NEXAFS) deposited by magnetron sputtering, and Cu powder (used for MS). These different sample geometries were selected to be compatible with the different measurement techniques adopted: Cu foil is an easily workable geometry for AP-XPS which can be cleaned by sputtering and annealing cycles; Cu thin films can be deposited with low enough thickness to allow X-rays to penetrate in the AtmP-NEXAFS cell; Cu powder offers higher surface area to generate a measurable mass signal change in MS. Whilst this leads to some variation in surface morphology and structure, as we characterize and discuss further below, all can be considered polycrystalline, with an ensemble of different Cu surfaces and grain boundaries being observed for the probe sizes used herein. Comparisons between the data collected with the different catalyst variants are therefore made within this context.

To investigate the morphology of the different catalyst variants, scanning electron microscopy (SEM) was performed. All samples were exposed to air before being loaded into the SEM. The powder and thin film were measured using a Carl Zeiss Merlin field emission gun SEM, while the foil was measured using a Carl Zeiss EVO SEM with a tungsten electron source. Electron backscatter diffraction (EBSD) was performed with an acceleration voltage of 20 kV, using a Bruker Quantax e-Flash1000 system.

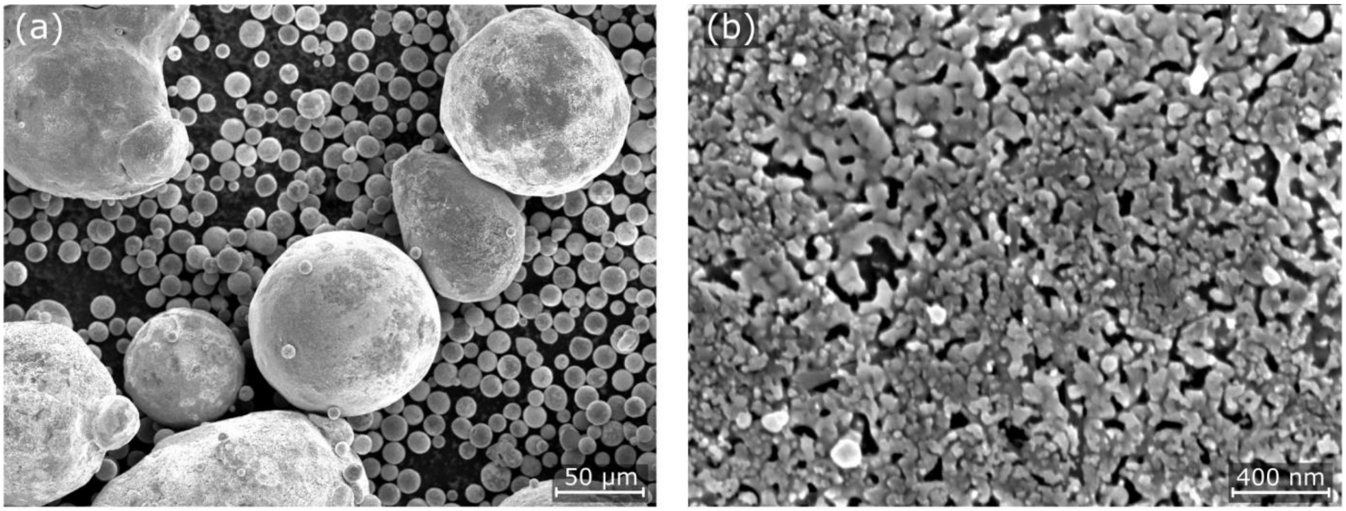

Figure S1: SE micrographs of annealed Cu powder at low (a) and high (b) magnification (see scale bars). Both images were measured at 5 kV accelerating voltage.

Figure S1 shows a SE micrograph of the annealed powder (the annealing procedure is described above). The low magnification image in Figure S1(a) shows a bimodal distribution of Cu particles sizes, some having a diameter ~100 µm, while smaller particles have diameters <10 µm, consistent with the -100 mesh of the purchased powder (i.e. all particles have diameters ≤150 µm). The high magnification image in Figure S1(b) shows a somewhat porous surface, with grain sizes of tens to hundreds of nanometres. This is consistent with previous reports where the reduction of copper oxide yields a similar porous morphology, and thus a larger Cu surface area that exhibits many different surfaces.[1]

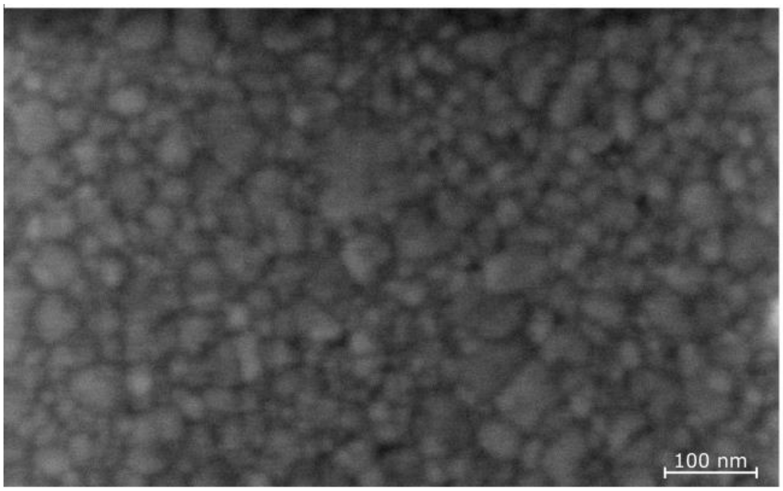

Figure S2: SE micrograph of annealed Cu thin film measured at a 2 kV accelerating voltage.

Figure S2 shows a SE micrograph of the sputter-deposited Cu(60 nm) thin film on SiN_x, following a similar surface treatment to that described in the main manuscript (275°C in 50 mbar of H_2 for 30 mins). Many small grains can be seen in the figure, with similar lateral dimensions to the film's thickness and comparable dimensions to those observed for the Cu powder. Some preferential <111> texture is expected for such a supported thin film annealed to this temperature, but the relatively small grain size and surface topography apparent in the SEM image indicates a high density of low coordination sites associated with vicinal surfaces and grain boundaries.

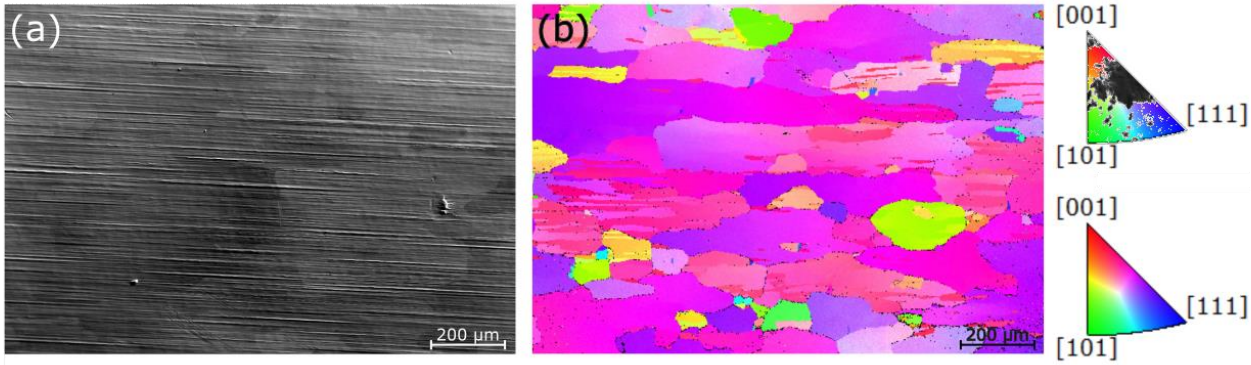

Figure S3: (a) SE micrograph of Cu foil (25 μm) following Ar^+-ion sputtering and annealing (300 °C), acquired at 20 kV accelerating voltage in secondary electron mode. (b) EBSD map of Cu foil shown in a. The inverse pole figure color code with and without grain orientation data plotted is displayed to the right. Note that SEM and EBSD are not taken in the same region.

Figure S3(a) shows a SE micrograph of the Cu foil after sputtering and annealing. Clear rolling striations can be seen across the sample surface associated with manufacture of the Cu foil. The grain structure is also visible through channeling contrast, with lateral dimensions comparable to the foil thickness. Figure S3(b) shows a EBSD map of grain orientations in the direction of the surface normal, with the inverse pole figure color code also shown. A variety of orientations are present, but some preferential <112> texture is apparent, consistent with the rolling texture induced during the cold-rolling used in foil manufacture. The topography associated with the rolling striations on the Cu foil mean that even individual grains will not have a single well-defined surface orientation but contain a variety of vicinal surfaces which will be simultaneously probed in our APXPS measurements. A high density of low coordination sites is therefore expected on the surface of the Cu foil. We note the much lower annealing temperatures used in our work compared to other studies that consider a Cu<111> surface to form by repeated sputter annealing of polycrystalline Cu.[2]

Chemical Characterization of Cu Powder

Cu powder was purchased from Alfa Aesar (-100 mesh (≤149 μm), 99% purity) and used for MS, providing a higher surface area than the Cu thin film/foil and thereby increasing the yield of reaction products. Here, we replicate a similar pretreatment procedure to that used for the MS measurements in order to investigate the initial reduction behavior of the powder and thereby facilitate direct comparison with the foil and thin film samples used for AP-XPS and AtmP-NEXAFS respectively. Cu powder was loaded into a ceramic boat and placed in a glass tube with inlet and outlet valves within a tube furnace. The inlet of the glass tube was supplied with a H_2:Ar (5%:95%) gas mixture. The outlet was connected to the exhaust through a bubbler, with gas flow monitored by the extent of bubble formation. The furnace temperature was increased at 5°C/min to 270°C and then held for an hour before cooling. The inlet and outlet valves of the work tube were subsequently sealed and transferred into a glovebox, before the powder was recovered. The difference between the as received and annealed powder can be seen in the photograph in Figure S4. The as received material is a dull-brown color initially, which becomes a brighter pink-orange after annealing, characteristic of metallic copper.

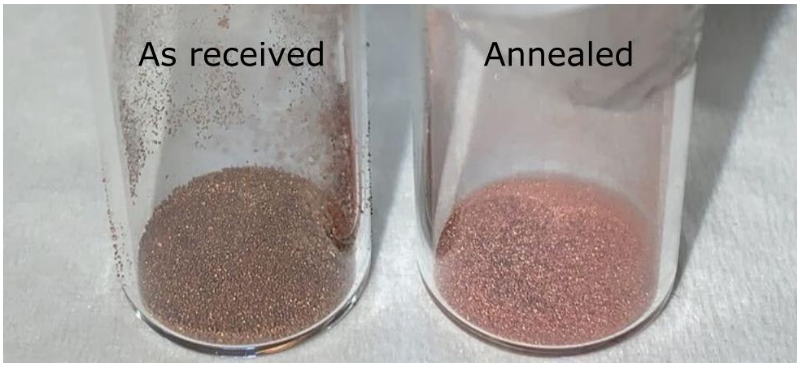

Figure S4: Cu powder as received (left) and annealed to 275 °C in 5%:95% H2:Ar (right).

In order to probe the surface chemical state of the powder after this procedure, the annealed powder was inertly transferred into a VersaProbe III XPS system, which has a base pressure of ~2×10^{-9} mbar and a monochromated Al Kα anode source (energy resolution ~0.5 eV at pass energy 23 eV determined from fitting the Fermi edge of a gold foil). This data is shown in Figure S5 along with measurements of the as received material.

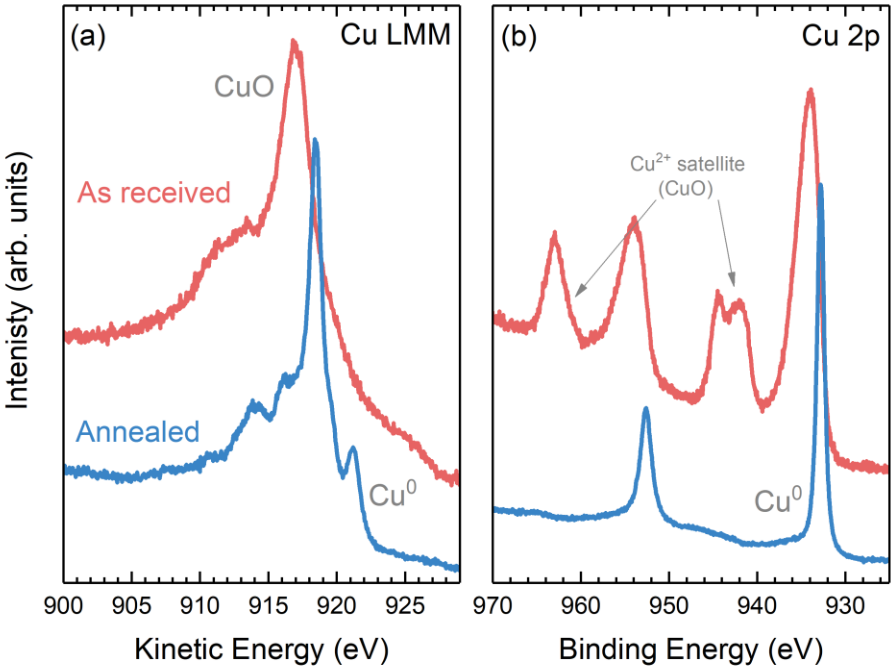

Figure S5: (a) Cu LMM and (b) Cu 2p of the as received and annealed Cu powder.

The as received powder shows the spectral features of Cu^{2+} (CuO) in both the Cu LMM and Cu 2p regions, with the Cu LMM spectrum in Figure S5(a) showing a prominent peak at ~917.0 eV and a broad but defined shoulder towards lower kinetic energy. Meanwhile the Cu $2p_{3/2}$ peak is located at ~933.9 eV in Figure S5(b) with pronounced satellite structure apparent at higher binding energies above both peaks of the

doublet. The Cu LMM of the annealed powder is consistent with metallic Cu, having a sharper main peak at lower energy than in either oxide and with more structure in its spectral shape, whilst Cu^{+} has a very broad main peak which merges with its shoulder to a greater extent. The Cu 2p region shows a much weaker satellite structure compared to the as received powder and the main Cu $2p_{3/2}$ peak is found to be at slightly lower binding energy of ~932.8 eV. Although it is challenging to distinguish Cu^{+} and Cu^{0} from the Cu 2p spectrum, when taken with the Cu LMM, this confirms the surface of the powder is metallic following annealing. Therefore, these data show that the pretreatment process for the MS measurements yields the same surface chemical state on the powder as for the other catalyst systems investigated in this study.

<u>Mass Spectrometry of CH_3OH:</u>

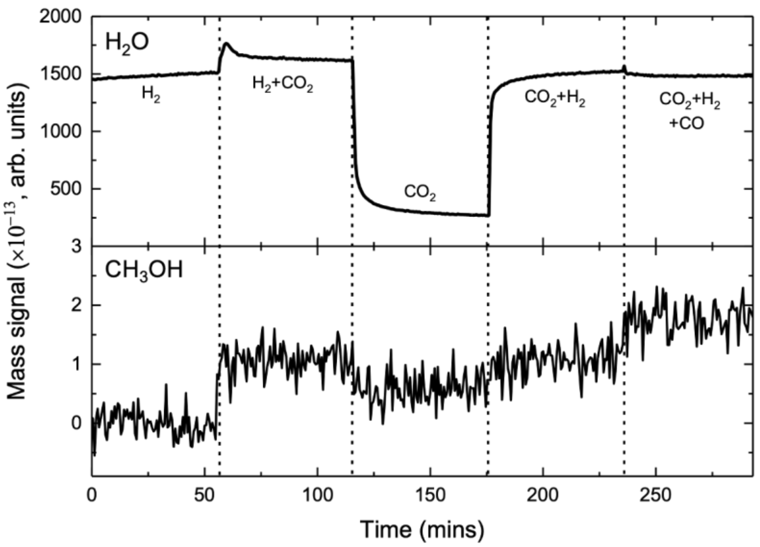

Figure S6: Time evolution of mass signals for H_2O and CH_3OH at 200 °C during exposure to H_2 (20 sccm), H_2 (20 sccm) and CO_2 (20 sccm), CO_2 (20 sccm), H_2 (20 sccm) and CO_2 (20 sccm), and H_2 (20 sccm) and CO_2 (20 sccm) and CO (10 sccm).

References:

(1) Unutulmazsoy, Y.; Cancellieri, C.; Lin, L.; Jeurgens, L. P. H. Reduction of Thermally Grown Single-Phase CuO and Cu2O Thin Films by in-Situ Time-Resolved XRD. *Appl. Surf. Sci.* **2022**, *588* (February), 152896.

(2) Favaro, M.; Xiao, H.; Cheng, T.; Goddard, W. A.; Crumlin, E. J. Subsurface Oxide Plays a Critical Role in CO2 Activation by Cu(111) Surfaces to Form Chemisorbed CO2, the First Step in Reduction of CO2. *Proc. Natl. Acad. Sci. U. S. A.* **2017**, *114* (26), 6706–6711.
